# Structural and Functional Changes Are Related to Cognitive Status in Wilson’s Disease

**DOI:** 10.3389/fnhum.2021.610947

**Published:** 2021-02-25

**Authors:** Sheng Hu, Chunsheng Xu, Ting Dong, Hongli Wu, Yi Wang, Anqin Wang, Hongxing Kan, Chuanfu Li

**Affiliations:** ^1^Center for Biomedical Engineering, University of Science and Technology of China, Hefei, China; ^2^School of Medical Information Engineering, Anhui University of Chinese Medicine, Hefei, China; ^3^Medical Imaging Center, First Affiliated Hospital of Anhui University of Chinese Medicine, Hefei, China

**Keywords:** Wilson’s disease, functional connectivity, diffusion tensor imaging, functional magnetic resonance imaging, visual association cortex, association fibers, limbic fibers

## Abstract

Patients with Wilson’s disease (WD) suffer from prospective memory (PM) impairment, and some of patients develop cognitive impairment. However, very little is known about how brain structure and function changes effect PM in WD. Here, we employed multimodal neuroimaging data acquired from 22 WD patients and 26 healthy controls (HC) who underwent three-dimensional T1-weighted, diffusion tensor imaging (DTI), and resting state functional magnetic resonance imaging (RS-fMRI). We investigated gray matter (GM) volumes with voxel-based morphometry, DTI metrics using the fiber tractography method, and RS-fMRI using the seed-based functional connectivity method. Compared with HC, WD patients showed GM volume reductions in the basal ganglia (BG) and occipital fusiform gyrus, as well as volume increase in the visual association cortex. Moreover, whiter matter (WM) tracks of WD were widely impaired in association and limbic fibers. WM tracks in association fibers are significant related to PM in WD patients. Relative to HC, WD patients showed that the visual association cortex functionally connects to the thalamus and hippocampus, which is associated with global cognitive function in patients with WD. Together, these findings suggested that PM impairment in WD may be modulated by aberrant WM in association fibers, and that GM volume changes in the association cortex has no direct effect on cognitive status, but indirectly affect global cognitive function by its aberrant functional connectivity (FC) in patients with WD. Our findings may provide a new window to further study how WD develops into cognitive impairment, and deepen our understanding of the cognitive status and neuropathology of WD.

## Introduction

Wilson’s disease (WD) is an inherited disorder of copper metabolism characterized pathologically by the deposition of copper in many organs, particularly the brain and liver, resulting in numerous clinical symptoms (Bandmann et al., 2015; Czlonkowska et al., 2018). Cognitive impairment is relatively frequent in WD patients, mainly with the frontal lobe syndrome and subcortical dementia (Lang, 1989; Lang et al., 1990). Cognitive impairment greatly affects the quality of life of WD patients. After suffering from cognitive impairment, WD patients will firstly experience prospective memory (PM) impairment. PM, which is a memory component most closely related to the planning or goal-making of daily activities, is defined as the future plans or intention of memory (Arnold et al., 2015). PM include event-based PM (EBPM) and time-based PM (TBPM), which are required to perform purposeful behaviors in the presence of specific target events and goals (McDaniel and Einstein, 2011). Recent studies have reported that PM impairment is associated with gray matter (GM) loss in the basal ganglia (BG) and structural changes in frontal and occipital whiter matter (WM) (Dong et al., 2016, 2019). However, how structural and functional changes affect PM of WD is still unknown.

Advances in neuroimaging have greatly improved the understanding of pathophysiology in WD, especially brain alterations in association with neurological symptoms. Structural brain MRI studies have revealed that widespread alternations in extrapyramidal system nuclei (putaminal and thalamic softening cavitation, caudate nucleus shrinkage, spongy change in the midbrain, and atrophy in thalamus) served as the key features to distinguish the neurological symptoms of WD (Koziæ et al., 2003; Stezin et al., 2016; Zou et al., 2019). Another study reported that GM atrophy in the frontal, parietal, and temporal lobes had a positive correlation with duration of disease (Stezin et al., 2016). One study investigating WM microstructure reported a decreased fractional anisotropy (FA) of frontal and occipital WM, bilateral internal capsules, midbrain, and pons in WD patients with neurological symptoms (Jadav et al., 2013). A recent resting state functional magnetic resonance imaging (RS-fMRI) study using machine learning to classify brain networks discovered that aberrant brain networks in WD patients are associated with severity of clinical symptoms (Jing et al., 2019). However, PM as crucial clinical features in WD patients was not taken into account by the majority of neuroimaging studies.

Association fibers (including superior longitudinal fasciculus, inferior longitudinal fasciculus, inferior fronto-occipital fasciculus, and uncinate fasciculus) and limbic system fibers (including cingulum) are reported to relate to brain function, regarding cognitive control, working memory, and perception of visual space (Delano-Wood et al., 2012; Metzler-Baddeley et al., 2012; Martino and De Lucas, 2014; Motomura et al., 2014; Metoki et al., 2017; Herbet et al., 2018). Therefore, association and limbic system fiber might contribute to PM impairment in patients with WD. Taking this assumption into account, the association and limbic system fibers are structures of interest in the current study.

Here, we hypothesize that WM changes in association and limbic system fibers and GM changes are associated with PM impairment in WD, and furtherly aberrant changes in GM volume would cause brain function deficits that are associated with cognitive status in patients with WD. In order to solve these problems, the present study collected structural magnetic resonance imaging (sMRI) and diffusion tensor imaging (DTI) to investigate structural changes (including the cortical and subcortical GM, and association and limbic fibers of WM) in WD patients compared with healthy controls (HC). In order to identify whether aberrant GM volume changes have an influence on brain function, we further collect RS-fMRI to evaluate functional connectivity (FC) changes in patients with WD. Finally, whether structural and functional changes are associated with PM impairment of WD was investigated by correlation analysis.

## Materials and Methods

### Subjects

Twenty-two native Chinese-speaking WD patients (12 men, 10 women; age: 22.36 ± 8.09 years; age range: 10–36 years) and 26 HC (14 men, 12 women; age: 22.18 ± 7.67 years; age range: 12–35 years) were recruited at the First Affiliated Hospital of Anhui University of Chinese Medicine (AUCM). After comprehensive clinical interviews evaluated by an neurologist expert and a trained neuropsychologist, WD patients based on the diagnosis of clinical symptoms of Kayser–Fleischer (KF) rings, neuroimaging findings, and abnormal copper metabolism were included, they were excluded if they had any other neurological or psychiatric disorder. HC had no history of head injury, neurological, psychiatric disorder, or concomitant medical disorder. All WD patients enrolled in the study have been in stable drug therapy for 2 weeks. Research approval was obtained from the Human Research Committee of the First Affiliated Hospital of AUCM and written informed consent was signed by all subjects before the enrollment. Table 1 summarizes the clinical and demographic characteristics of all subjects enrolled in the present study.

**TABLE 1:** Clinical features of patients and healthy controls.

	**WD (*N* = 22)**	**HC (*N* = 26)**
Gender (male/female)	12/10	14/12
Age (years)	10–36 (22.36 ± 8.09)	12–35 (22.18 ± 7.67)
Education (years)	7–12 (8.73 ± 1.24)	6–14 (9.73 ± 2.55)
Handedness	22 right-handed	26 right-handed
Duration (years)	1–10 (5.25 ± 3.03)	-
MMSE	25–28 (26.59 ± 0.85)	-
EBPM	3–6 (4.73 ± 0.88)	-
TBPM	2–4 (2.68 ± 0.99)	-
KF ring	22 WD with a KF ring	-
SCU (ug/dL)	15–58 (34.34 ± 14.43)	-
SCP (mg/dL)	2–10 (4.74 ± 2.29)	-

*WD, Wilson’s disease; HC, healthy controls; MMSE, Mini-Mental State Examination; EBPM, event-based prospective memory; TBPM, time-based prospective memory; N, number; KF, Kayser–Fleischer; SCU, serum copper; SCP, serum ceruloplasmin.*

### Neuropsychological Assessment

The neuropsychological assessment of WD patients was performed by an experienced neuropsychologist. WD patients were evaluated by cognitive function measured with the Mini-Mental State Examination (MMSE), and PM. Full details are presented in the supplementary information. The neuropsychological scale is provided in Table 1.

### MRI Acquisition

Magnetic resonance imaging data were acquired using a 3.0-Tesla MR system (Discovery MR750, General Electric, Milwaukee, WI, United States) with an eight-channel high-resolution radio-frequency head coil. Sagittal 3D T1-weighted images were acquired using a T1-3D BRAVO sequence (repetition time (TR)/echo time (TE), 8.16 ms/3.18 ms; flip angle, 12°; matrix, 256 mm × 256 mm; field of view (FOV), 256 mm × 256 mm; slice thickness, 1 mm) with 170 axial slices with no gap. RS-fMRI images were acquired using a gradient-echo single-shot echo planar imaging sequence (TR/TE, 2,000 ms/30 ms; FOV, 220 mm × 220 mm; matrix 64 mm × 64 mm; flip angle, 90°; slice thickness, 3 mm) with 185 volumes. Diffusion tensor images were acquired by an echo-planar imaging (EPI) sequence (TR/TE, 6,000 ms/81.7; matrix, 128 mm × 128 mm; FOV, 256 mm × 256 mm; slice thickness, 3 mm) with a *b* value of 2,000 s/mm^2^ and 64 gradient diffusion directions evenly distributed on a sphere. During scanning, all subjects were instructed to remain motionless and to keep their eyes closed.

### MRI Analysis

Structural MRI data were analyzed with FSL version 5.0.2^1^, and RS-fMRI data were analyzed with AFNI version 19.2.21^2^. The flowchart of data processing is presented in Supplementary Figure 1.

#### GM Volume Measurement

Gray matter volume measurement was performed using FSL-VBM (Ashburner and Friston, 2000). 3D T1-weighted images were first taken for all the subjects. FSL Automated Segmentation Tool (FAST) was employed to highlight the GM from the 3D T1-weghted image. The segmented GM parietal volume images were then normalized to the MNI152 standard space using the linear image registration (Jenkinson and Smith, 2001) (FLIRT) and non-linear registration (Smith et al., 2004) (FNIRT) tool box. The normalized images were further averaged and flipped along the x-axis to create a study-specific GM template. All native GM images were subsequently non-linearly registered to the study-specific template and modulated for the contraction due to the non-linear component of the transformation by dividing them by the Jacobian of the warp field. The modulated GM images were finally smoothed with an isotropic Gaussian kernel with a sigma of 3 mm. The study-specific GM template was divided into 110 regions of interest (ROIs) based on the Harvard-Oxford cortical and subcortical probabilistic atlases, which included the cerebral cortex and BG but excluded the cerebellum. GM volume for each ROI was measured for further statistical analysis.

Group differences of GM volumes were measured using two-sample *t* test analysis with age as the covariate, and a false discovery rate corrected (FDR) for multiple comparisons. The correlations between GM volumes of significantly different ROI and neuropsychological symptoms were evaluated by linear-regression analysis, FDR was used for multiple comparisons.

#### Functional Connectivity Analysis

**FIGURE 1:**
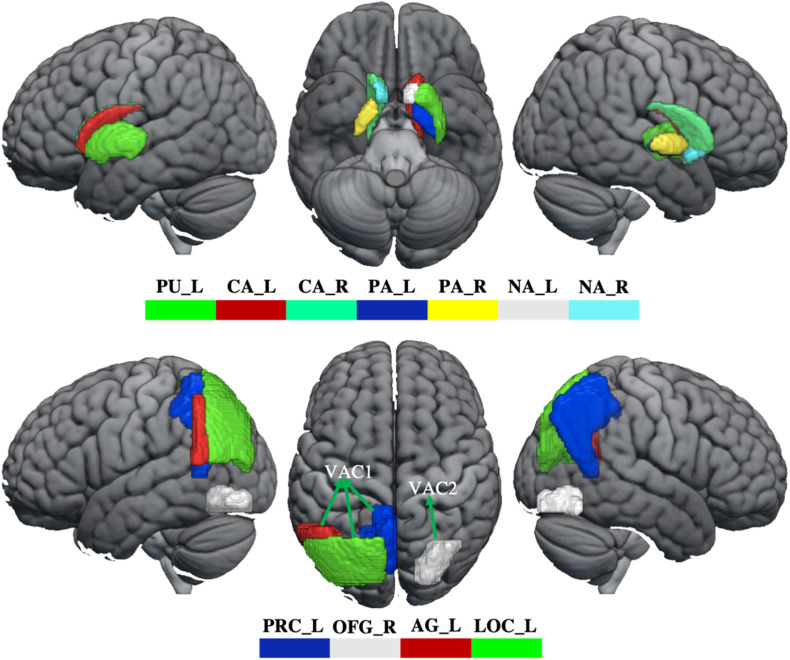
The abnormity of GM volume in WD. The top panel shows that GM volume reduces in the basal ganglia. The bottom panel shows the GM volume changes in the visual association cortex. The GM volume increased in VAC1 and decreased in VAC2. PU_L, left putamen; CA_L, left caudate; CA_R, right caudate; PA_L, left pallidum; PA_R, right pallidum; NA_L, left accumbens; NA_R, right accumbens; PRC_L, left precuneus; OFG_R, right occipital fusiform gyrus; AG_L, left angular gyrus; LOC_L, lateral occipital cortex; VAC1, visual association cortex 1; VAC2, visual association cortex 2.

In order to evaluate how GM volume changes have effects on brain function, the aberrant brain regions (BG, visual association cortex) were selected as seeds to perform seed-based FC analysis. The visual association cortex (VAC) was divided into two seeds, including VAC1 (GM volume increased in left angular gyrus, lateral occipital cortex, and precuneus) and VAC2 (GM volume reduced in right occipital fusiform gyrus). Therefore, three seeds (BG, VAC1, and VAC2, Figure 1) were selected for FC analysis. For each participant, the Pearson correlation coefficient between the mean time series of seeds and the time series of every voxel across the whole brain was calculated, and the coefficients were further converted to a z-value using a Fisher r-to-z transformation to improve the normality (Biswal et al., 1995). Therefore, each participant acquired a BG-based FC map, VAC1-based FC map, and VAC2-based FC map.

A group-level analysis was applied to identify the FC differences between WD and HC. Group-wise whole brain analysis (AFNI package, version 19.2.21, see text footnote 2) of the belief-photo contrasted with a voxel-wise *p* threshold of 0.001 and cluster-wise threshold of 0.01. The relation between FC of WD patients in significantly different brain regions and neuropsychological symptoms were calculated by linear-regression analysis and the results were corrected by FDR for multiple comparisons.

In order to evaluate relationships between FC and GM volumes, correlation analysis was performed in regions with GM volumes that had significant differences between WD and HC, and the results were corrected by FDR for multiple comparisons.

#### WM Tracks

Data preprocessing for the diffusion tensor images was carried using a standard pipeline. The diffusion tensor data were skull-stripped using the BET tool (Smith, 2002), and corrected for distortions caused by eddy currents and movements using the eddy tool (Graham et al., 2016). The diffusion tensor was assessed using the DTIFIT toolbox to acquire FA, axial (AD), mean (MD), and radial diffusivities (RD) maps. Using a “seed” method, the reconstructions of association fibers [superior longitudinal fasciculus (SLF), inferior longitudinal fasciculus (ILF), inferior fronto-occipital fasciculus (IFO), and uncinate fasciculus (UNC)] and limbic fibers [cingulate gyrus part of cingulum (CGC) and the parahippocampal part of cingulum (CGH)] were acquired by performing fiber tracking in a native diffusion tensor space with a probabilistic tractography algorithm (Behrens et al., 2007) (PROBTRACKX) as implemented in FDT, which was based on the Bayesian estimation of diffusion parameters (BEDPOSTX) obtained using sampling techniques (Hernández et al., 2015). For each track, the average FA, AD, MD, and RD were extracted in native space.

Group differences of WM were calculated using two-sample *t* test analysis with age as the covariate, and the false discovery rate corrected (FDR) for multiple comparisons. The correlations between metrics (FA, MD, AD, and RD) of significantly different WM and neuropsychological symptoms were evaluated by linear-regression analysis, FDR was used for multiple comparisons.

## Results

### WM Tracks

**FIGURE 2:**
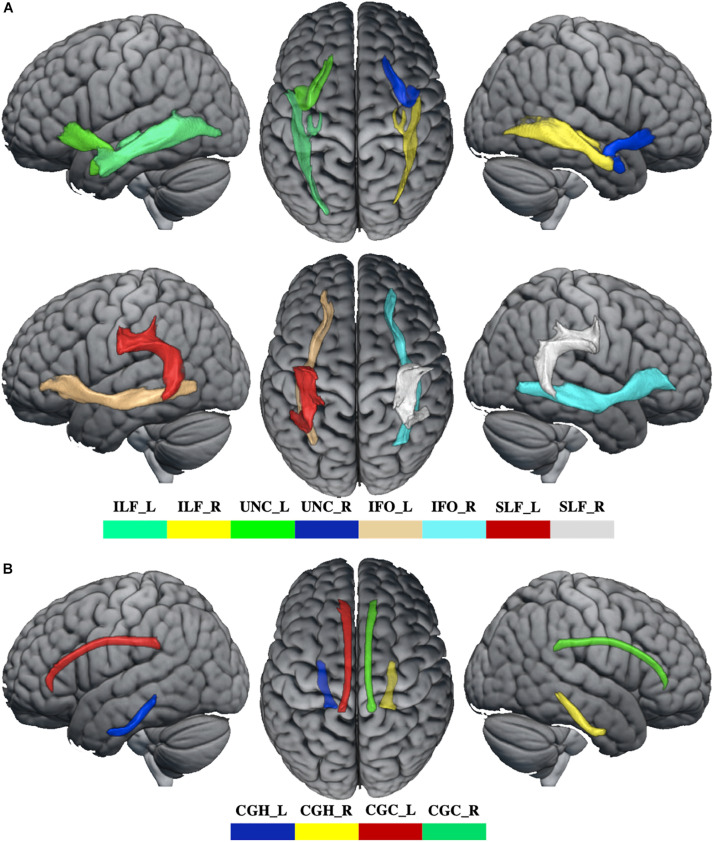
The abnormal WM tracks in WD. **(A)** WM tracks of association fibers. **(B)** WM tracks of limbic system fibers. ILF_L, left inferior longitudinal fasciculus; ILF_R, right inferior longitudinal fasciculus; UNC_L, left uncinate fasciculus; UNC_R, right uncinate fasciculus; IFO_L, left inferior fronto-occipital fasciculus; IFO_R, right inferior fronto-occipital fasciculus; SLF_L, left superior longitudinal fasciculus; SLF_R, right superior longitudinal fasciculus; CGH_L, parahippocampal part of cingulum; CGH_R, right parahippocampal part of cingulum; CGC_L, cingulate gyrus part of cingulum; CGC_R, right cingulate gyrus part of cingulum.

**FIGURE 3:**
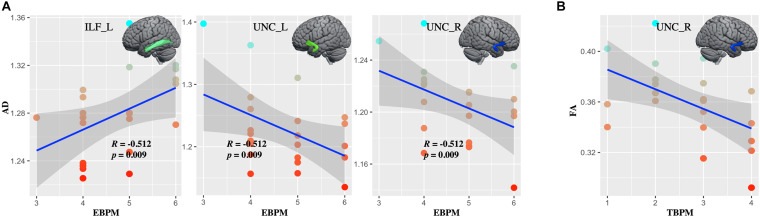
The correlations between diffusion metrics and clinical symptoms. **(A)** Axial diffusivity (AD) in the left ILF and bilateral UNC correlated with the score at EBPM. **(B)** Fractional anisotropy (FA) in right UNC correlated with the score at TBPM. ILF_L, left inferior longitudinal fasciculus; UNC_L, left uncinate fasciculus; UNC_R, right uncinate fasciculus.

Whiter matter tracks of WD patients were widely impaired in association and limbic system fibers (Figure 2 and Supplementary Table 2). Specifically, compared with healthy controls, WD patients showed increased MD and RD in all association and limbic tracks, increased AD in the left CGH, bilateral IFO, SLF, and UNC, and decreased FA in all limbic system fibers, bilateral SLF and UNC. WM tracks had significant correlations with neuropsychological symptoms. Specifically, WD patients showed that AD of the left ILF (*r* = 0.451, *p* = 0.035), UNC (*r* = −0.449, *p* = 0.036) and right UNC (*r* = −0.437, *p* = 0.042) had significant correlations with EBPM (Figure 3A), and that FA of the right UNC (*r* = −0.516, *p* = 0.014) had significant correlations with TBPM (Figure 3B). There were no group differences of head motion between HC and WD (*t* = −0.5579, *p* = 0.5796) (Supplementary Figure 2A). It suggested that head motion has no influence on results.

### GM Volumes

Compared with healthy controls, WD patients showed GM volume loss in the BG, including bilateral caudate, pallidum and accumbens, left putamen, and also in the visual association cortex, located in the right occipital fusiform gyrus (OFG). However, compared with healthy controls, WD patents revealed increased GM volumes in the visual association cortex, including left angular gyrus (AG), lateral occipital gyrus (LOC), and precuneus (Figure 1 and Supplementary Table 1). There were no significant correlations between GM volume and neuropsychological symptoms in patients with WD.

### Functional Connectivity

**FIGURE 4:**
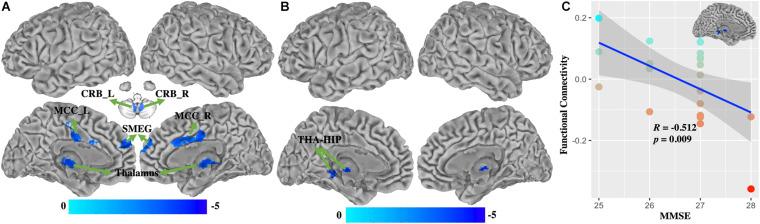
The functional connectivity changes between WD and HC. **(A)** Functional connectivity decreased in the basal ganglia. **(B)** Functional connectivity decreased in VAC1. **(C)** Functional connectivity between VAC1 and THA-HIP was correlated with the score of MMSE in WD patients. THA-HIP, thalamus and hippocampus; CRB_L, left cerebellum; CRB_R, right cerebellum; MCC_L, left middle cingulate cortex; SMEG, superior medial frontal gyrus; MMSE, Mini-Mental State Examination.

Compared with healthy controls, WD patients demonstrated that FC of BG (Figure 4A and Supplementary Table 3) was decreased in the bilateral cerebellum, left thalamus, middle cingulate cortex (MCC), and superior medial frontal gyrus (SMEG), and that FC of VAC1 (Figure 4B and Supplementary Table 4) was decreased in the left thalamus and hippocampus (THA-HIP). No significant FC of VAC2 differences were found between groups. FC between the visual association cortex and THA-HIP was negatively correlated (*r* = −0.512, *p* = 0.009) with MMSE (Figure 4C). There were no group differences of head motion between HC and WD (*t* = −1.447, *p* = 0.1546) (Supplementary Figure 2B). It suggested that head motion had no influence on results.

### Correlations Between FC and GM Volumes

Based on the BG-based FC map of WD, significant correlation between FC value and gray volume (GV) value was found in BG (*R* = 0.75, *P* < 0.001), and no significant correlations were found in VAC1 (*R* = 0.07, *P* = 0.77) and VAC2 (*R* = −0.089, *P* = 0.69) (Supplementary Figure 3A). Based on the VAC1-based FC map, no significant correlations between FC value and GV value were found in VAC1 (*R* = −0.146, *P* = 0.52), VAC2 (*R* = −0.063, *P* = 0.78), and BG (*R* = 0.08, *P* = 0.74) (Supplementary Figure 3B).

## Discussion

What are the fundamental brain alternations that lead to cognitive impairment in WD patients? Although prior studies have reported that WD can cause frontal lobe syndrome, or subcortical dementia, direct evidence of the correlation between brain alternations and neuropsychology is still lacking. In this study, we used a multimodal approach to analysis brain structural and functional changes to show that WM changes in association fibers are associated with PM, and that GM atrophy in the visual association cortex leads to aberrant FC that effects global cognitive function in WD. These multiparametric MRI finds may have implications in understanding the neural mechanisms underlying cognitive status in WD.

In this study, half of the patients had mild neurological symptoms and the other half had no symptoms. Clinical symptoms of the WD patients were not very serious compared to other studies (Stezin et al., 2016). In line with previous neuroimaging studies in WD, we observed similar GM atrophy in the BG (including bilateral caudate, pallidum and accumbens, and left putamen nuclei). GM atrophy is reported to be due to edema, neuronal necrosis, gliosis, demyelination, or cystic degeneration of neurons due to copper overload or hepatic dysfunction (Kim et al., 2006; Bruha et al., 2011; Beinhardt et al., 2014). No significant correlations were found between GM atrophy of BG and PM, which is inconsistent with a previous study (Dong et al., 2016). However, many previous studies have revealed the widespread alternations in BG which mainly lead to extrapyramidal symptoms such as tremors, ataxia, and dystonic syndrome (Ala et al., 2007; Algin et al., 2010). The possible interpretation is that PM impairment is easily masked by other symptoms (Lorincz, 2010) that did not manifest in structural damage in BG in the present study. Interesting, based on the BG-based FC map, we also found that GM volume had significant correlation with FC in BG. This indicated that GM volume atrophy in BG had a significant effect on its function, and further affected the clinical symptoms in WD. Compared to healthy controls, no widespread cortical atrophy was found except in the right OFG; however, GM volumes increased in the visual association cortex (including left AG, LOC, and precuneus) in WD patients. Astrocytes are first impaired by copper accumulation which can cause astrogliosis, followed by cellular swelling, and then the upregulation of the synthesis of metallothionein to increase storage capacity for copper (Scheiber and Dringen, 2011). Due to the lower severity of neuropathology in WD patients, copper toxicity does not have an extensive impact on the cerebral cortex. Therefore, cortical atrophy does not widely occur in WD patients. Several forms of astrocytes exist in the central nervous system including fibrous (in WM), protoplasmic (in GM), and radial (Figley and Stroman, 2011; Khakh and Sofroniew, 2015) astrocytes. Copper accumulation can result in an increase in astrocyte numbers so that increased GM volumes in the visual association cortex may be associated with increased protoplasmic astrocytes. Another possible interpretation is that medication induces cortical changes to generate a compensatory response to structural impairments in patients with WD. Several prior studies have pointed out that neuroanatomical alternations in patients, such as schizophrenia and Parkinson’s disease are associated with drug therapy (Navari and Dazzan, 2009; Fusar-Poli et al., 2013). However, there are no significant correlations between the value of GM volumes in the visual association cortex and the score of PM and MMSE. The visual association cortex is not thought to play a general role in memory function (Rosen et al., 2017). Previous studies have demonstrated that the visual association cortex along with the hippocampus play a role in association memory formation (Rosen et al., 2017). This may indicate that structural damages in the visual association cortex do not have a direct effect on PM and MMSE.

The FC analysis showed that compared with healthy controls, patients’ FC of BG was decreased in large-scale networks, including the bilateral cerebellum, right thalamus and MCC, and left SMEG. FC of BG did not correlate with cognitive performance. Previous studies have confirmed that MRI abnormalities in BG are responsible for extrapyramidal or neurological symptoms in WD (Bandmann et al., 2015). The behaviors that have the most striking relationships with FC of BG were limb movements, motivation and, decision making, although measures of cognitive function also showed relationships (Albin et al., 1989; Kreitzer and Malenka, 2008). Taken together, these results suggest that FC changes in BG are important in producing extrapyramidal or neurological disorders in WD patients, instead of PM and global cognitive function impairment. We also demonstrated that FC of VAC1 was decreased in the left THA-HIP. FC between VAC1 and THA-HIP is significantly related to global cognitive function in patients with WD. Connectivity from the thalamus to the visual cortex was investigated to confirm its involvement in visual cognition. Specifically, evidence suggested that the pulvinar, the largest thalamic nucleus, receives input from structures such as the retina and superior colliculus which critically shape the functional organization of the visual cortex, which contributes to visual cognition (Bridge et al., 2016). VAC1 is dorsal stream of the visual system which is proposed to be involved in the perception and interperception of spatial relationships, guidance of actions, and learning tasks that coordinate the body in the space (Galletti and Fattori, 2018). For another, the thalamus is functionally connected to the hippocampus with respect to spatial memory and spatial sensory datum (Stein et al., 2000; Aggleton et al., 2010). A recent study has reported that coactivation of the association visual cortex and hippocampus mediate association memory performances (Rosen et al., 2018). Therefore, these results may suggest that the diminished ability of perception spatial information from the visual cortex impaired FC between VAC1 and THA-HIP, and further affected global cognitive function in WD patients.

Structural changes of WM were widely observed in association and limbic fibers with preservation of the cortex, which can be explained by observation of gliosis and spongiosis in pathology studies (Meenakshi-Sundaram et al., 2002). These results may reflect a reduction of WM microstructural organization in brain locations as the results were compatible with axonal loss and/or demyelination (Jadav et al., 2013). WM changes in bilateral UNC were clinically relevant to the PM scales in WD patients. The UNC, linking anterior portions of the temporal lobe with the inferior frontal lobe, has a role in some types of learning and memory (Alm et al., 2015). In line with a multiple sclerosis study, it suggested that PM deficits are related to alternation of UNC (Pardini et al., 2014). The mean AD of ILF was positively correlated with EBPM. This is an intriguing finding, suggesting that a lower AD may be more prone to develop into PM deficits. Several studies have shown that altered ILF is associated with episodic memory in patients with cognitive decline (Hodgetts et al., 2017; Gao et al., 2019). ILF are thought to connect the temporal gyrus to the fusiform gyrus, occipital gyri, lingual gyrus, and ceneus (Latini et al., 2017). This may imply that increased or reduced GM volume in the visual association cortex is influenced by WM changes in ILF. In this study, we selected regions with GM differences as seed to perform seed-based FC analysis, and found that aberrant FC between the visual association cortex and THA-HIP was associated with global cognitive function. These may indicate that WM and GM have an effect on each other, and further affect brain function in patients with WD. The UNC and ILF may be structural signatures characterizing the PM deficits in WD patients.

In summary, using a multimodal MRI approach, the present study provides a comprehensive picture of structural and functional brain alternations in WD patients relative to healthy controls. On one hand, WM changes in association fibers are related to PM impairment in WD patients. On the other hand, WD patients manifested GM volume decreases in BG and increases in the visual association cortex that is abnormally connected to THA-HIP with respect to impaired global cognitive function in patients with WD. These findings suggested that PM impairment in WD may modulate aberrant WM in association fibers, and that GM volume changes in the association cortex have no direct effect on cognitive status, but indirectly affect global cognitive function by its aberrant FC in patients with WD. Our findings may provide a new window to further study how WD develops into cognitive impairment, and deepen our understanding of the cognitive status and neuropathology of WD.

### Limitations

There are several limitations in the present study. The longer scanning time of fMRI makes the results of FC more stable. Patients with WD did not have enough patience to complete the long-term experiment when compared to healthy controls. Therefore, we limited fMRI scanning time to 6 min, which is less than the common scanning time of fMRI. In the future study, we will increase the scanning time appropriately for fMRI data so that the results of FC will be more stable. In the current study, we investigated how WM alternations impact on PM, and how GM volume atrophy causes aberrant FC which has an impact on cognitive status in patients with WD. However, we do not know how brain function is mediated by WM and GM and further affect the cognitive status in patients with WD. In the future, mediation analysis will be performed in our next study to investigate this problem and provide more useful and interesting results.

## Data Availability Statement

The original contributions presented in the study are included in the article/Supplementary Material, further inquiries can be directed to the corresponding author/s.

## Ethics Statement

The studies involving human participants were reviewed and approved by Human Research Committee of The First Affiliated Hospital of Anhui University of Chinese Medicine. Written informed consent to participate in this study was provided by the participants’ legal guardian/next of kin.

## Author Contributions

CL and TD designed the current study. SH and CX drafted the manuscript. SH, CX, and AW performed the experiments. SH and HW analyzed the data. SH, YW, and HK revised the manuscript. All authors read and approved the final manuscript.

## Conflict of Interest

The authors declare that the research was conducted in the absence of any commercial or financial relationships that could be construed as a potential conflict of interest.

## Footnotes

1 http://fsl.fmrib.ox.ac.uk/fsl

2 https://afni.nimh.nih.gov
