# Supplementary material for: Structural and Functional Changes Are Related to Cognitive Status in Wilson’s Disease
Source: Front Hum Neurosci. 2021 Feb 25;15:610947. doi: 10.3389/fnhum.2021.610947 (PMC7947794; doi:10.3389/fnhum.2021.610947)
Supplement: Supplementary file 1 [file Table_1.DOCX]

**Supplementary information: Neuropsychological assessment (MMSE, EBPM and TBPM); Supplementary Tables 1-4;** **Supplementary Figure 1-2; References.**

**Neuropsychological assessment**

In cognitive neuropsychology, prospective memory is defined as the future plans or intentions of memory and a memory component that is most closely related to daily activities(Arnold et al., 2015). The neuropsychological assessment of WD patients was performed by an experienced neuropsychologist. Global cognitive functioning measured with the Mini-mental State Examination (MMSE), event-based prospective memory (EBPM) and time-based prospective memory (TBPM) were evaluated for WD patients.

***MMSE.*** The MMSE was administrated to estimate the global functions, including temporal and spatial orientation, short-term memory, calculation, language, and visuo-spatial skill(Folstein et al., 1975).

***EBPM.*** Before the experiment, patients were informed the following instructions: tapping the desk whenever they found the two animal words (target event) during the task and providing their telephone numbers after the tests were finished (this instruction won’t be reminded any more when the tests were finished). Specifically, patients were instructed to perform a word selection task, using 30 question cards each of which were printed 12 high frequency Chinese content words. Ten of the 12 words belonged to one category, and the remaining two words belonged to another category. Subjects were instructed to read the two words that belonged to the category that differed from the other 10 words from each card. The target events for the EBPM task occurred on the 5^th^, 10^th^, 15^th^, 20^th^, 25^th^, and 30^th^ card of the word selection task. The patients’ performance in the word selection task was recorded. One point was awarded when patients correctly response to a target event (total six target events). Another two points were awarded for remembering to provide their telephone number after the test. The maximum score in EBPM task was 8 (Cheng et al., 2018).

***TBPM.*** Patients were told to tap the desk at the target time points of 5, 10, 15min during the task. Patients were instructed to use a digital clock to check the time by themselves. At the beginning of the test, the clock was set to 0:00:00, and the test is stopped when the clock indicated 17 minutes. After the clock was started, patients were asked for performing the number selection task, which included 100 cards. On each card, 12 different two-digit numbers were printed. Patients were asked to choose the smallest and largest number in the cards. The exact time at which the subjects responded by tapping the desk was recorded. If patients responded from 10 seconds before to 10 seconds after the target time, patients will get 2 points. One point was awarded if patients were responded from 30 seconds before to 30 seconds after the target time. The maximus score of the TBPM task was 6 (Cheng et al., 2018).

| **Regions** | **Side** | **Wilson’s Disease Group (N=22)** | | **Healthy Control Group (N=26)** | | **Test**  **WD vs HC** | |
| --- | --- | --- | --- | --- | --- | --- | --- |
|  |  | **Mean** | **SD** | **Mean** | **SD** | **T** | **p** |
| AG | L | 0.5412 | 0.0928 | 0.4776 | 0.0677 | 2.7407 | 0.0432 |
| LOC | L | 0.4692 | 0.0557 | 0.4330 | 0.0349 | 2.7451 | 0.0466 |
| Precuneus | L | 0.5594 | 0.0573 | 0.5170 | 0.0425 | 2.9387 | 0.0341 |
| OFG | R | 0.6043 | 0.0691 | 0.6901 | 0.0855 | -3.7772 | 0.0045 |
| Accumbens | L | 0.4744 | 0.0889 | 0.6777 | 0.1185 | -6.6200 | < 0.001 |
| Caudate | L | 0.3629 | 0.1188 | 0.5399 | 0.0697 | -6.4097 | < 0.001 |
| Putamen | L | 0.2714 | 0.0983 | 0.3507 | 0.0671 | -3.3066 | 0.0157 |
| Pallidum | L | 0.0621 | 0.0169 | 0.0799 | 0.0120 | -4.2662 | 0.0012 |
| Caudate | R | 0.3789 | 0.1159 | 0.5482 | 0.0664 | -6.3277 | < 0.001 |
| Pallidum | R | 0.0779 | 0.0216 | 0.0951 | 0.0153 | -3.2075 | 0.0182 |
| Accumbens | R | 0.4510 | 0.0952 | 0.6146 | 0.0925 | -6.0230 | < 0.001 |

**Supplementary Table 1.** GM volumes in WD patients and healthy controls. Only significant differences of GM volume in cortical regions between WD and healthy controls were reported. Group differences were measured with two-sampled t test and the results were corrected by false-discovery rate for multiple comparisons. Abbreviation: AG, Angular gyrus; LOC, Lateral occipital gyrus; OFG, Occipital fusiform gyrus; WD, Wilson’s disease; HC, Healthy controls; L, left; R, right.

| **Regions** | **Side** | **Wilson’s Disease Group (N=22)** | | **Healthy Control Group (N=26)** | | **Test**  **WD vs HC** | | **Wilson’s Disease Group (N=22)** | | **Healthy Control Group (N=26)** | | **Test**  **WD vs HC** | |
| --- | --- | --- | --- | --- | --- | --- | --- | --- | --- | --- | --- | --- | --- |
|  |  | **Mean** | **SD** | **Mean** | **SD** | **T** | **p** | **Mean** | **SD** | **Mean** | **SD** | **T** | **p** |
| **FA Values (×10^-1^)** | | | | | | | | **MD Values (×10^-4^)** | | | | | |
| CGC | L | 3.38 | 0.805 | 4.49 | 0.508 | -3.516 | 0.003 | 8.75 | 0.482 | 8.14 | 0.217 | 5.793 | <0.001 |
| CGC | R | 3.62 | 0.750 | 4.50 | 0.469 | -5.003 | <0.001 | 8.56 | 0.553 | 7.87 | 0.192 | 5.981 | <0.001 |
| CGH | L | 2.28 | 0.268 | 2.57 | 0.238 | -4.054 | <0.001 | 9.87 | 1.098 | 9.05 | 0.381 | 3.543 | 0.001 |
| CGH | R | 2.43 | 0.263 | 2.73 | 0.249 | -4.055 | <0.001 | 9.38 | 0.776 | 8.82 | 0.328 | 3.368 | 0.0017 |
| IFO | L | 4.05 | 0.202 | 4.15 | 0.198 | -1.694 | 0.116 | 8.90 | 0.506 | 8.36 | 0.173 | 5.186 | <0.001 |
| IFO | R | 4.12 | 0.201 | 4.21 | 0.231 | -1.493 | 0.142 | 8.43 | 0.234 | 8.12 | 0.163 | 5.304 | <0.001 |
| ILF | L | 3.97 | 0.232 | 4.11 | 0.225 | -2.128 | 0.052 | 8.78 | 0.300 | 8.54 | 0.191 | 3.371 | 0.0017 |
| ILF | R | 3.95 | 0.171 | 4.05 | 0.243 | -1.545 | 0.141 | 8.55 | 0.215 | 8.42 | 0.168 | 2.276 | 0.027 |
| SLF | L | 3.70 | 0.204 | 3.85 | 0.163 | -2.850 | 0.013 | 8.17 | 0.358 | 7.78 | 0.171 | 4.953 | <0.001 |
| SLF | R | 3.36 | 0.243 | 3.53 | 0.200 | -2.661 | 0.018 | 8.53 | 0.527 | 7.98 | 0.210 | 4.880 | <0.001 |
| UNC | L | 3.52 | 0.259 | 3.71 | 0.186 | -2.859 | 0.013 | 8.82 | 0.640 | 8.10 | 0.179 | 5.534 | <0.001 |
| UNC | R | 3.59 | 0.299 | 3.79 | 0.231 | -2.554 | 0.021 | 8.56 | 0.335 | 8.22 | 0.184 | 4.449 | <0.001 |
| **AD Values (×10^-3^)** | | | | | | | | **RD Values (×10^-4^)** | | | | | |
| CGC | L | 1.22 | 0.056 | 1.21 | 0.059 | 0.302 | 0.763 | 7.03 | 0.869 | 6.15 | 0.396 | 4.679 | <0.001 |
| CGC | R | 1.21 | 0.068 | 1.22 | 0.053 | -0.554 | 0.635 | 6.81 | 0.860 | 5.72 | 0.387 | 5.800 | <0.001 |
| CGH | L | 1.21 | 0.103 | 1.15 | 0.045 | 2.602 | 0.021 | 8.74 | 1.140 | 7.82 | 0.412 | 3.878 | <0.001 |
| CGH | R | 1.17 | 0.072 | 1.14 | 0.055 | 1.638 | 0.162 | 8.21 | 0.822 | 7.51 | 0.294 | 4.019 | <0.001 |
| IFO | L | 1.30 | 0.052 | 1.24 | 0.030 | 4.852 | <0.001 | 6.85 | 0.526 | 6.33 | 0.198 | 4.743 | <0.001 |
| IFO | R | 1.24 | 0.029 | 1.21 | 0.026 | 3.854 | <0.001 | 6.42 | 0.273 | 6.12 | 0.224 | 4.242 | <0.001 |
| ILF | L | 1.28 | 0.034 | 1.26 | 0.029 | 1.531 | 0.177 | 6.78 | 0.352 | 6.49 | 0.239 | 3.396 | 0.0015 |
| ILF | R | 1.24 | 0.033 | 1.23 | 0.025 | 0.810 | 0.506 | 6.61 | 0.216 | 6.45 | 0.247 | 2.287 | 0.027 |
| SLF | L | 1.15 | 0.033 | 1.11 | 0.025 | 3.853 | <0.001 | 6.52 | 0.409 | 6.09 | 0.183 | 4.777 | <0.001 |
| SLF | R | 1.15 | 0.044 | 1.11 | 0.023 | 4.969 | <0.001 | 7.01 | 0.591 | 6.43 | 0.260 | 4.506 | <0.001 |
| UNC | L | 1.23 | 0.065 | 1.15 | 0.029 | 5.046 | <0.001 | 7.10 | 0.676 | 6.37 | 0.193 | 5.288 | <0.001 |
| UNC | R | 1.21 | 0.029 | 1.18 | 0.024 | 3.076 | 0.007 | 6.81 | 0.436 | 6.42 | 0.251 | 3.896 | <0.001 |

**Supplementary Table 2.** Diffusion Tensor MRI metrics of whiter matter tracks in WD patients and healthy controls. Group differences of WM were measured with two-sampled t test and the results were corrected by false-discovery rate for multiple comparisons. Abbreviations: CGC, cingulate gyrus part of cingulum; CGH, parahippocampal part of cingulum; SLF, superior longitudinal fasciculus; ILF, inferior longitudinal fasciculus; IFO, inferior fronto-occipital fasciculus; UNC, uncinate fasciculus; WD, Wilson’s disease; FA, fractional anisotropy; AD, axial diffusivity; MD, mean diffusivity; RD, radial diffusivity; HC, Healthy controls; L, left; R, right.

| **Regions** | **Side** | **MNI Coordinate** | | | **Voxel** | **z value**  **peak** |
| --- | --- | --- | --- | --- | --- | --- |
|  |  | **x** | **y** | **z** |  |  |
| Thalamus | R | 10 | -10 | 6 | 1254 | -4.94 |
| Cerebellum | L | -6 | -56 | -46 | 581 | -4.86 |
| MCC | R | 4 | -8 | 36 | 551 | -4.30 |
| SMEG | L | -6 | 58 | 26 | 445 | -4.77 |
| Cerebellum | R | 10 | -56 | -42 | 277 | -4.99 |

**Supplementary Table 3.** Group differences between WD patients and healthy controls in functional connectivity of basal ganglia. Group differences in functional connectivity of basal ganglia were measured with two-sampled t test and the whole brain correction was performed via Monte Carlo simulations (3dCluSim, AFNI package, version 19.2.21) at a voxel-wise height threshold of p < 0.001 and a cluster size threshold of 180 contiguous voxels. The results were presented in coordinate of peak value. Abbreviations: MCC, middle cingulate cortex; SMEG, superior medial frontal gyrus; L, left; R, right.

| **Regions** | **Side** | **MNI Coordinate** | | | **Voxel** | **z value**  **peak** |
| --- | --- | --- | --- | --- | --- | --- |
|  |  | **x** | **y** | **z** |  |  |
| THA-HIP | L | -10 | -21 | 8 | 535 | -4.79 |

**Supplementary Table 4.** Group differences between WD patients and healthy controls in functional connectivity of visual association cortex. Group differences in functional connectivity of basal ganglia were measured with two-sampled t test and the whole brain correction was performed via Monte Carlo simulations (3dCluSim, AFNI package, version 19.2.21) at a voxel-wise height threshold of p < 0.001 and a cluster size threshold of 180 contiguous voxels. The results were presented in coordinate of peak value. Abbreviations: THA-HIP, Thalamus and hippocampus; L, left.


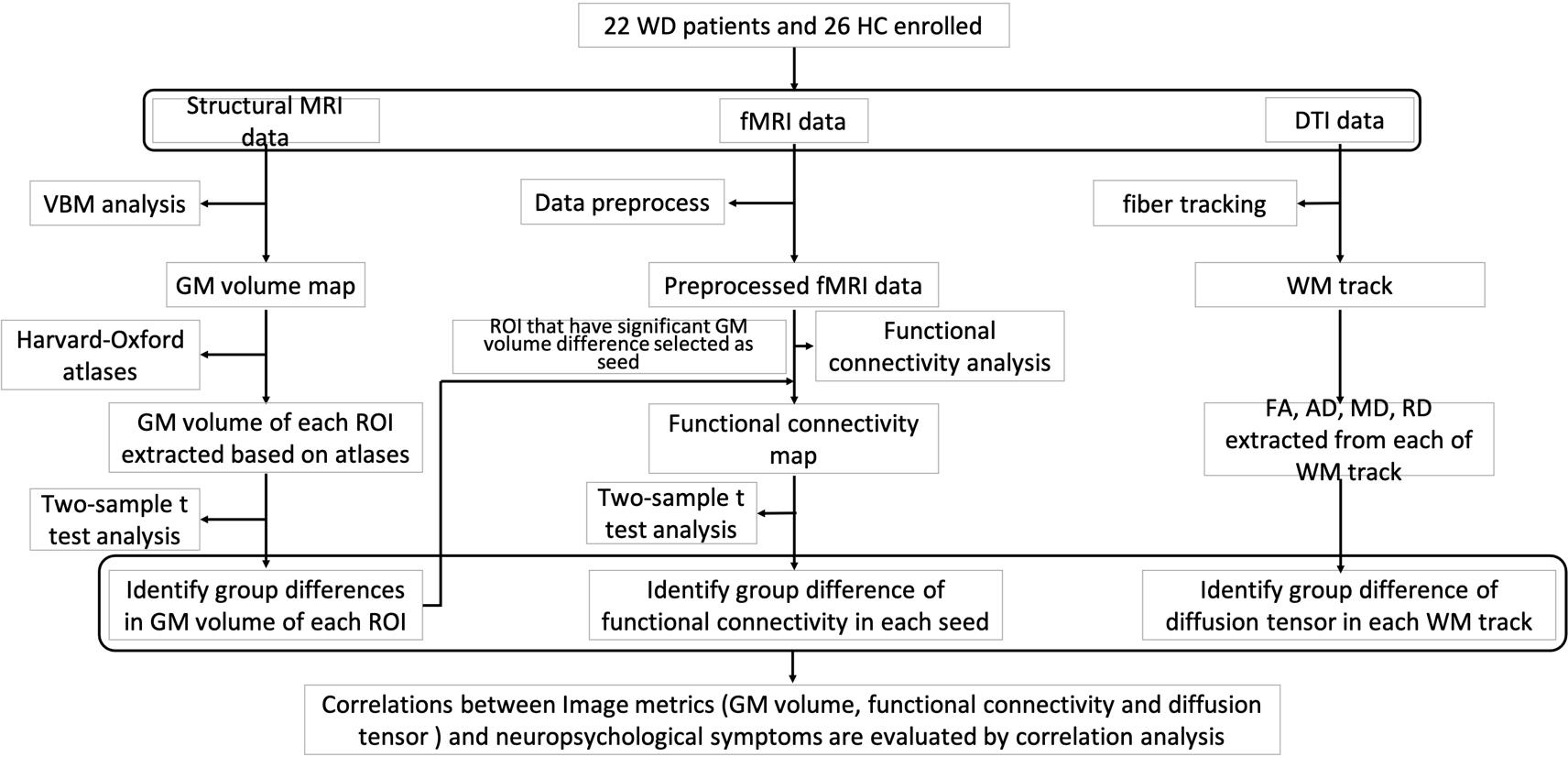


**Supplementary Figure 1.** Data processing flowchart


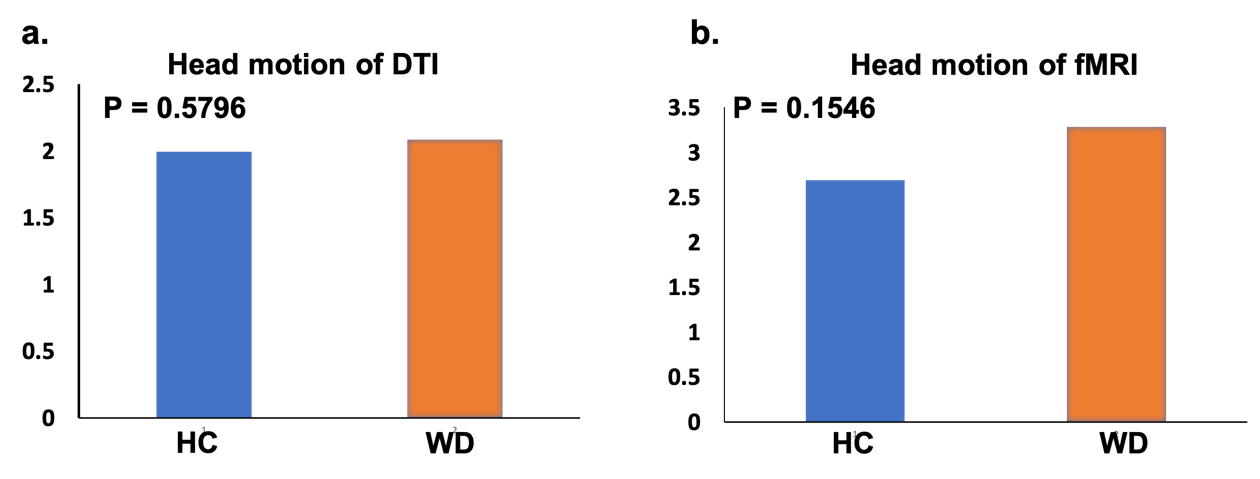


**Supplementary figure 2.** The graph display the frame-wised displacement of head motion. There are no group differences between two groups.

**
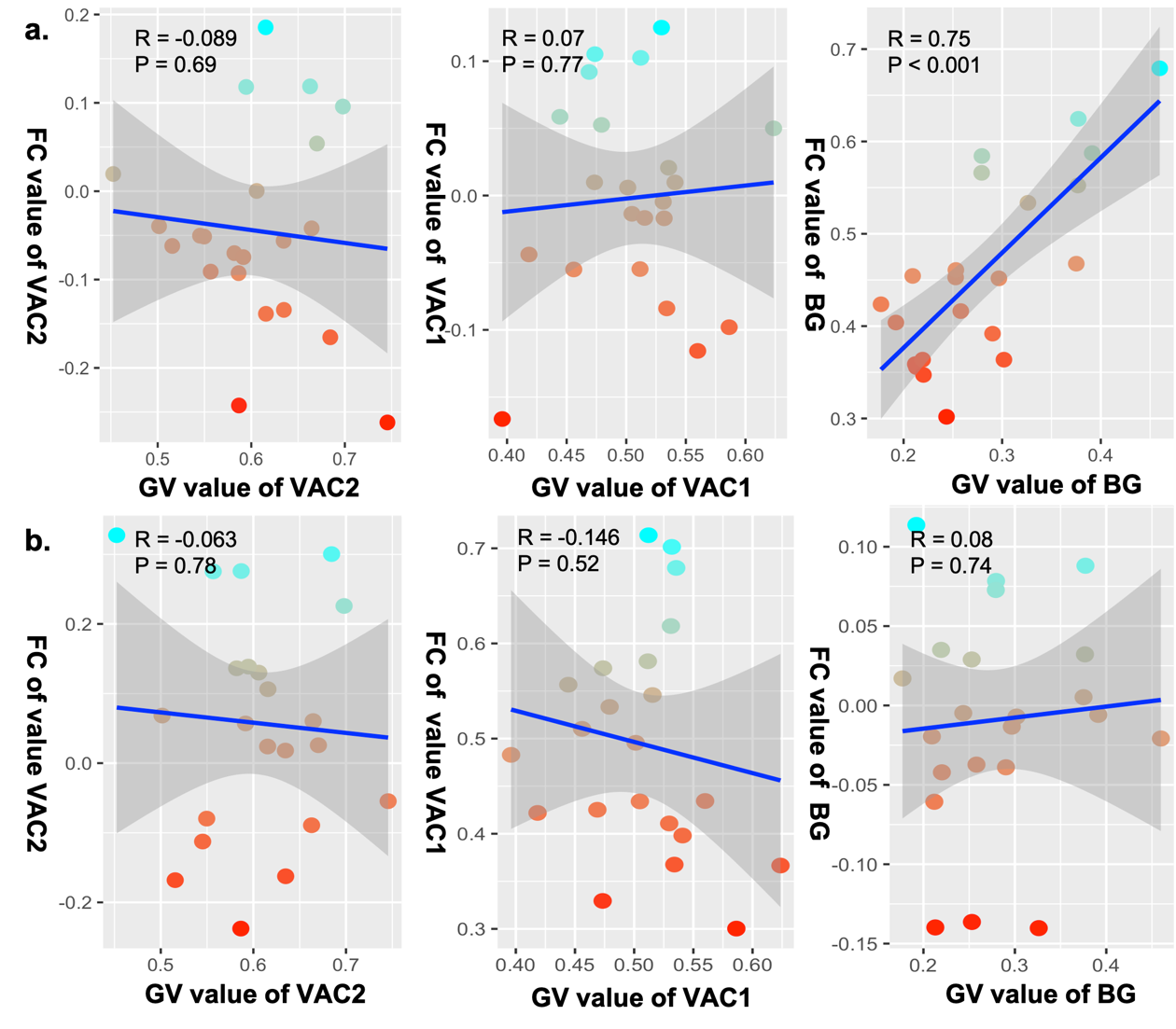
**

**Supplementary figure 3.** Correlations between functional connectivity and gray volume. The value of FC and GV were extracted from regions that volumes of gray matter have significant differences between WD and HC. a. Correlation between FC value which was extracted based on BG-based FC map and GV value. b. Correlation between FC value which was extracted based on VAC1-based FC map and GV value. Abbreviations: GV, gray volume; FC, functional connectivity.

**References**

Arnold, N.R., Bayen, U.J., Böhm, M.F., 2015. Is prospective memory related to depression and anxiety? A hierarchical MPT modelling approach. Memory (Hove, England) 23, 1215-1228.

Cheng, H., Chen, H., Lv, Y., Chen, Z., Li, C.-S.R., 2018. Prospective memory impairment following whole brain radiotherapy in patients with metastatic brain cancer. Cancer Medicine 7, 5315-5321.

Folstein, M.F., Folstein, S.E., McHugh, P.R., 1975. "Mini-mental state". A practical method for grading the cognitive state of patients for the clinician. J Psychiatr Res 12, 189-198.
